# Tocilizumab Use in a Chronic Hemodialysis Patient for the Management of COVID-19-Associated Pneumonia and Acute Respiratory Distress Syndrome

**DOI:** 10.1155/2020/8829309

**Published:** 2020-11-23

**Authors:** Sudeendra Gupta, Rakesh Madhyastha, Fadi Hamed, Maher Balkis, Wasim El Nekidy, Nizar Attallah

**Affiliations:** ^1^Medical Sub-specialities Institute, Cleveland Clinic Abu Dhabi, Abu Dhabi, UAE; ^2^Critical Care Institute, Cleveland Clinic Abu Dhabi, Abu Dhabi, UAE; ^3^Department of Pharmacy, Cleveland Clinic Abu Dhabi, Abu Dhabi, UAE

## Abstract

Novel coronavirus disease 2019 (COVID-19) is a highly infectious, rapidly spreading viral disease. As of writing this article, there are over 4.4 million people affected by COVID-19, and unfortunately, 300,000 have succumbed to the infection. In this article, we address a particularly more susceptible group of the population of end-stage renal disease (ESRD) patients on dialysis who may potentially benefit from being treated with tocilizumab. The use of tocilizumab has not been reported widely in ESRD patients on dialysis to treat COVID-19. In this case report, we describe a patient with ESRD on hemodialysis who was admitted to the intensive care unit, with severe pneumonia secondary to COVID-19 infection. This patient was treated with tocilizumab 400 mg intravenous and had a favorable outcome with no apparent adverse events.

## 1. Introduction

Coronaviruses are RNA viruses and typically cause symptoms similar to the common cold. But two coronaviruses—severe acute respiratory syndrome coronavirus (SARS-CoV-2) and Middle East respiratory syndrome coronavirus (MERS-CoV)—have been associated with severe pneumonia, acute respiratory failure, and death [1, 2]. In late 2019, deadly infections related to a novel coronavirus (SARS-CoV-2) appeared in China and has been rapidly spreading worldwide [3].

An epidemiological survey shows that patients with underlying conditions such as diabetes mellitus, hypertension, and cardiovascular disease or the elderly are more susceptible to acquire COVID-19 infection [2]. Hemodialysis (HD) patients are particularly vulnerable to infection and may exhibit greater variations in clinical symptoms and infectivity [3]. Patients with end-stage renal disease (ESRD) on HD are susceptible to infection mainly because of more exposure to the healthcare system and personnel on top of having multiple comorbidities [3–5].

In the absence of quality evidence, several agents that showed previous activity against SARS-CoV-1 and MERS-CoV have emerged. Hydroxychloroquine, an antimalaria drug commonly used to treat chronic inflammatory diseases, has shown in vitro activity against SARS-CoV-2, but that benefit has not been consistently demonstrated across large clinical trials and recently lost its approval [6, 7]. Lopinavir/ritonavir showed in vitro activity, and few case reports demonstrated success but without support from clinical trials [8]. Interferon-*α* and -*β* were used in China to treat SARS-CoV-2 [8]. Remdesivir showed activity against SARS-CoV-2 in recent clinical trials and received FDA approval for that [9, 10]. Favipiravir, another antiviral, used to treat influenza in Japan, showed activity against SARS-CoV-2 [6]. Finally, anticytokines immunomodulators such as tocilizumab antagonizes the interleukin-6 (IL-6) receptors and consequently inhibits the cytokine storm. In a small clinical trial from China, patients with severe SARS-CoV-2 infection significantly improved with tocilizumab and were successfully discharged [6, 11]. The drug can cause elevation of liver enzymes and uric acid; however, little is known about the use of this drug in patients on renal replacement therapy.

We report a patient with ESRD on HD who was admitted to the intensive care unit (ICU), with severe pneumonia secondary to COVID-19 infection. This patient was treated with tocilizumab and had a favorable outcome with no apparent adverse events.

## 2. Case Report

Our patient was a 39-year-old male with a history of ESRD (of unknown underlying cause) treated with maintenance HD twice a week for the past 4.5 years. The patient presented to the emergency department (ED) with a 4-day history of fever and cough and a 1-day history of breathlessness. The patient was admitted to the ICU from the emergency department due to acute hypoxic respiratory failure secondary to severe pneumonia. He was having breathlessness (normal jugular venous pressure) with no peripheral edema; however, he had extensive crackles bilaterally. The O_2_ saturation was 70% on room air. He was tachypneic and in respiratory distress.

Furthermore, investigations revealed severe azotemia and lymphopenia with normal leucocyte counts. His arterial blood gas (ABG) was indicative of acute respiratory distress syndrome (ARDS) with PaO_2_/FiO_2_ < 200. Chest X-ray showed bilateral perihilar and basal infiltrates which rapidly worsened over the following 48 hrs with an increase in density and extensively involved the bilateral mid and upper zones.

**Figure 1:**
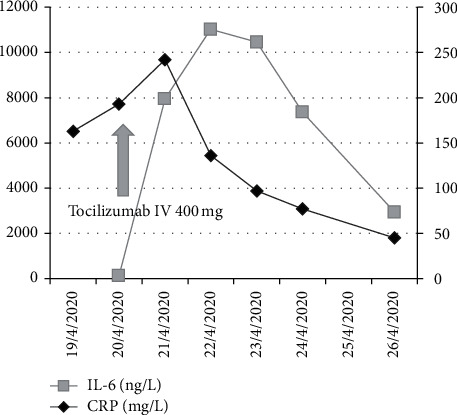
Effect of tocilizumab on inflammatory markers. IL-6, interleukin-6; CRP, *C*-reactive protein.

**Figure 2:**
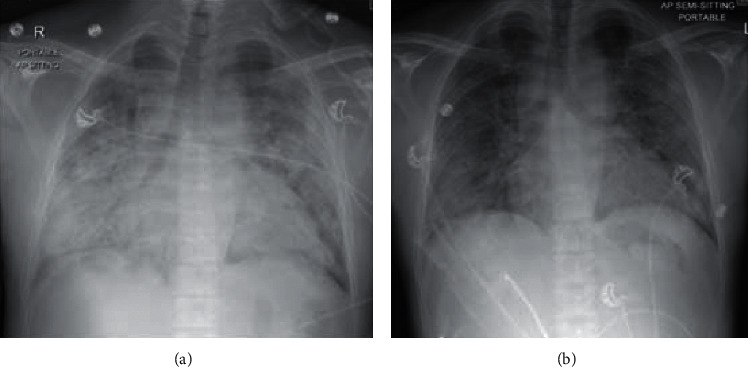
Chest X-ray AP view showing extensive bilateral perihilar and basal opacities on day 2 (a), which has improved significantly by day 4 as shown in (b).

The nasopharyngeal swab was positive for SARS-CoV-2 by real-time polymerase chain reaction (PCR). His inflammatory markers such as *C*-reactive protein (CRP), IL-6, and ferritin were significantly elevated (Figure 1). He was diagnosed with COVID-19-related bilateral pneumonia and ARDS. Noninvasive ventilation (NIV) was started. Hydroxychloroquine and regular HD with adequate ultrafiltration were initiated. On day 2 of admission, tocilizumab (IL-6 receptor antagonist) 400 mg IV one dose was given. On day 4, significant improvement occurred as shown by decreased breathlessness, decreased support of NIV and O_2_ requirements, and improvement in the bilateral chest infiltrates as demonstrated by chest X-ray (Figure 2). By day 7, the patient was transferred to the isolation ward (dedicated to patients with COVID-19) with minimal oxygen requirement (nasal cannula). He was then discharged home after finishing 14 days of isolation in a stable condition.

## 3. Discussion

We report this unique case as we expect several dialysis patients to have a more severe form of the infection. The IL-6 receptors were proven to play a key role in the cytokine storm, and blocking these receptors might potentially help patients with severe COVID-19 infections [12].

Several studies show that only 5% of COVID-19 patients have critical illness [13–15]. The mortality rate for ARDS patients with COVID-19 remains unclear but has relatively high variance as shown by an Italian study indicating critical care mortality up to 26% [14], while in the US the mortality was shown to be around 21% in the same population [15].

Management of patients with ARDS secondary to COVID-19 remains mainly supportive, although since the start of the pandemic, many other proposed treatments using antiviral medications, antimalarials, and anticytokine immunomodulators, such as the interleukin-6 (IL-6) receptor antagonist tocilizumab, have been utilized. Convalescent plasma has been used successfully in some instances [16].

Reports of using tocilizumab in an ESRD patient are scarce [15]. Tocilizumab is being used in patients to treat rheumatoid arthritis, but ESRD patients were excluded from the main studies [17, 18]. At the moment, the treatment of this infection in dialysis patients is supportive only. Unfortunately, the clinical trial of remedesivir, which is considered to be a novel agent against COVID-19, has excluded dialysis patients [9, 10, 19]. The risk profile for severe presentations of COVID-19 is reported to be concentrated in patients with comorbidities. Patients with ESRD are known to have multiple comorbidities. Unfortunately, most of those patients are excluded from the clinical trials, and we are left with no option but to use these medications to save this population' lives.

In a retrospective analysis of 21 patients used to treat COVID-19, tocilizumab effectively improved clinical symptoms and repressed deterioration of critically ill patients with COVID-19 [20]. Our patient was on chronic dialysis and was severely hypoxic on admission, with a rapidly deteriorating clinical condition for 48 hours before admission. After receiving tocilizumab, the picture completely inverted illustrating the role of this drug in the management of cytokine storm demonstrated by the reduction in all inflammatory markers (Figure 1 and Table 1) and improvement in patient's clinical picture.

IL-6 is a pleiotropic proinflammatory cytokine produced by a variety of cell types including T and B cells, lymphocytes, monocytes, and fibroblasts [21]. The overproduction of early response proinflammatory cytokines results in what has been described as a cytokine storm, leading to an increased risk of vascular hyperpermeability, multiorgan failure, and eventually death when the high cytokine concentrations are unabated over time [22]. IL-6 may play a key role in driving the inflammatory response that leads to morbidity and mortality and patients with COVID-19 who develop ARDS [22].

Tocilizumab is a recombinant humanized anti-human IL-6 receptor monoclonal antibody which inhibits IL-6-mediated signaling. The drug is recommended at a dose of 8 mg/kg intravenous either alone or in combination with corticosteroids. Our protocol recommends an intravenous dose of 4–8 mg/kg, up to a maximum of 400 mg in 250 ml of normal saline in one hour. The dose may be repeated in 12 hours. The maximum dose should not exceed 800 mg. The drug should not be administered to patients with tuberculosis or gastrointestinal perforation [23]. The drug, however, should not be initiated in a patient with severe neutropenia, thrombocytopenia, or elevated liver enzymes [11, 23, 24].

The pharmacokinetics of the drug reveals a volume of distribution of 6.4 L. The drug is known to have biphasic elimination from the circulation (nonrenal/nonhepatic). The drug has a long half-life between 11 and 13 days depending on the dose (4 mg/kg or 8 mg/kg, respectively). No formal study of the effect of renal impairment on the pharmacokinetics of tocilizumab was conducted. However, the drug manufacturer recommends no dose adjustment in patients with mild to moderate renal impairment. However, there is a lack of studies in patients with severe renal impairment or patients utilizing renal replacement therapies [23].

## 4. Conclusion

COVID-19 infection is affecting patients with different risk factors. Patients with ESRD are at higher risk for that mainly due to higher exposure to the healthcare system and have multiple comorbidities. Tocilizumab can be considered to treat sick patients with ESRD in the ICU with COVID-19 infection to combat the cytokine storm. It needs to be tested formally in a randomized controlled study.

## Figures and Tables

**Table 1:** Investigations.

	Day 1	Day 2	Day 3	Day 4	Day 5	Day 6	Day 7	Day 8	Day 9
BUN (mmol/L)	7.1	10.1	8.2	14.0	11.2	18.7	24.9	12.2	16.8
Creatinine (*μ*mol/L)	624	820	604	789	674	926	1121	710	931
Sodium (mmol/L)	135	132	137	133	136	131	129	134	133
Potassium (mmol/L)	3.9	4.7	4.9	5.1	4.2	4.1	4.3	4.3	4.6
Chloride (mmol/L)	92	91	93	92	93	89	87	92	90
Calcium (mmol/L)	2.21	1.95	2.05	2.06	2.11	2.27	2.26	2.27	2.32
Phosphorous (mmol/L)		0.77	2.1	2.16	1.73	1.98	2.07	1.79	2.12
Total bilirubin (*μ*mol/L)	3.6	4.3	5.0	3.6	3.7	4.4	3.6	4.0	3.3
Total protein/albumin (g/L)	69/38	68/36	66/34	64/32	64/34	67/35	66/33	67/35	65/35
AST (U/L)	87	111	125	69	115	133	61	46	34
ALT (U/L)	60	81	102	72	87	120	81	61	47
ALP (IU/L)	145	143	145	141	162	168	157	156	150
CRP (mg L)	163	193	242	136	97.5	77.9	57.9	45	30.2
IL 6 (ng/L)			7946	11024	10453	7360		2938	
Pro calcitonin (mcg/L)		18.9	30.1	28.28					
Ferritin (mcg/L)		6508	>8000	6656	5507	5152			
Hemoglobin (g/L)	90	90	91	81	81	80	82	85	85
Hematocrit	0.27	0.27	0.28	0.25	0.25	0.24	0.24	0.26	0.26
WBC (×10^9^L)	2.68	3.22	3.42	4.0	4.0	4.63	5.56	5.62	6.46
Neutrophil/lymphocyte (%)	84/12.9	68/27	62/26	70/16.8	66/16	65/18.6	66/18	63/20	60/20
Platelets (×10^9^/L)	117	118	110	116	126	124	137	161	174
	Day 1	Day 2		Day 3	Day 4	Day 5	Day 6	Day 7	
pH	7.4	7.49		7.40	7.42	7.43	7.43	7.44	
PaCO_2_ (kPA)	4.7	4.8		5.43	4.43	5.48	4.72	4.29	
PaO_2_ (kPA)	12.93	6.95		9.64	7.40	12.75	10.95	11.11	
HCO_3_ (mmol/L)	25	27		24	21	27	23	22	

## Data Availability

The data used to support the findings of this study are included within the article.
